# Viral-associated septic shock in pediatric pneumonia: a retrospective analysis of pathogen-specific risks and comparative clinical features with bacterial sepsis

**DOI:** 10.3389/fped.2025.1665453

**Published:** 2025-11-14

**Authors:** Ying Cheng, Kun Chen, Hui-ling Qian, Hong-bo Hu

**Affiliations:** 1 Department of Pediatrics, Maternal and Child Health Hospital of Hubei Province, Wuhan, China; 2 Department of Clinical Laboratory, Wuhan Ninth Hospital, Wuhan, China; 3 Department of Pediatrics, Wuhan Ninth Hospital, Wuhan, China; 4 Department of Clinical Laboratory, Maternal and Child Health Hospital of Hubei Province, Wuhan, China

**Keywords:** viral septic shock, pediatric pneumonia, pathogen-specific risk, clinical features, hospitalized children

## Abstract

**Background:**

This study aimed to determine the incidence of septic shock in hospitalized children with viral pneumonia, identify high-risk viral pathogens, and compare the clinical features between viral- and bacterial-associated septic shock cases.

**Methods:**

A retrospective study on viral respiratory infections in children hospitalized with pneumonia at two hospitals between 2022 and 2024 was conducted, with logistic regression used to assess the pathogen-specific risks.

**Results:**

Among 5,956 children with viral pneumonia, the incidence of septic shock was 1.06%, varying significantly by pathogen (*p* < 0.001). Influenza A (FluA) showed the highest incidence (3.70%) and was the strongest predictor of shock [odds ratio (OR) = 7.506], followed by respiratory syncytial virus (RSV) (2.24%; OR = 4.559). Compared to bacterial shock cases (*n* = 46), viral shock cases showed higher respiratory failure/acute respiratory distress syndrome (ARDS) rates (88.9% vs. 52.2%, *p* < 0.001) but they had shorter hospital stays (17.3 vs. 24.2 days, *p* = 0.026). FluA cases were more likely to have less infant involvement (*p* = 0.023), more neurologic compromise (35.7% vs. 8.7%, *p* = 0.040), and develop refractory shock (42.9% vs. 8.7%, *p* = 0.007) compared to bacterial cases. RSV showed higher respiratory failure (75.9% vs. 52.2%, *p* = 0.040) and lower coagulopathy (34.5% vs. 60.9%, *p* = 0.026) rates.

**Conclusions:**

FluA and RSV are the key viral pathogens that predispose pediatric pneumonia patients to septic shock. Rapid viral polymerase chain reaction testing enables early pathogen identification, facilitating antibiotic de-escalation when bacterial coinfection is unlikely and promoting precise, safe antimicrobial stewardship.

## 1 Introduction

Septic shock, a life-threatening condition characterized by circulatory collapse and multiorgan dysfunction secondary to severe infection, demonstrates considerable variation in clinical outcomes depending on the primary infection site (1–3). Among the patients in the emergency department, those with lower respiratory tract infections were at a much higher risk for developing sepsis and septic shock as well as have increased intensive care unit (ICU) admissions and higher 30-day death rates (1, 2, 4). Altogether, 38.2% of culture-negative septic shock cases reportedly are associated with LRTI (5), emphasizing the need for prompt identification and specialized management of respiratory infections to prevent the development of septic-related complications.

The role of identified respiratory viruses in pediatric septic shock cases remains controversial. Viral sepsis, defined as a systemic inflammatory response syndrome induced by viral infections and characterized by immune dysregulation and multiorgan dysfunction, has emerged as a critical yet understudied entity in pediatric medicine (6). Although the current pediatric sepsis guidelines predominantly emphasize bacterial etiologies, emerging evidence underscores the capacity of respiratory viruses to independently trigger sepsis and septic shock. This is evidenced by research on severe acute respiratory syndrome coronavirus 2 (SARS-CoV-2), revealing that a considerable proportion of critically ill pediatric patients with coronavirus disease 2019 (COVID-19) develop shock-like symptoms without any bacterial coinfections (7–9). Similarly, influenza-associated neuroinvasive complications have been linked to severe systemic inflammation and multiorgan failure, further highlighting this viral pathogenesis (10). However, the true prevalence of viral -associated septic shock is likely obscured by methodological limitations in both guidelines and epidemiological studies, including the systematic exclusion of viral cases (selection bias) and inadequate classification of pathogens. Consequently, a considerable gap persists in the existing guidelines, as they lack comprehensive recommendations for diagnosing and managing viral -associated septic shock in children.

The present study aimed to determine the incidence of septic shock occurs in children hospitalized with viral pneumonia, identify the viral pathogens associated with a higher risk of developing septic shock, and compare the clinical features between septic shock caused by viral infections and those caused by bacterial infections.

## 2 Methods

### 2.1 Inclusion criteria

The present retrospective cohort study was conducted at two hospitals from January 2022 to December 2024. We enrolled pediatric patients (aged >28 days to 15 years) hospitalized with a primary diagnosis of infectious pneumonia, which was established using a combination of clinical, radiographic, and microbiological criteria. Specifically, the diagnosis required the following criteria: (1) clinical and radiographic evidence: presence of clinical signs and symptoms (e.g., fever, cough, tachypnea, and auscultatory findings) and chest imaging findings (x-ray or computed tomography) consistent with pneumonia, as defined by the 2024 revised guidelines for the management of community-acquired pneumonia in children; and (2) microbiological confirmation: a confirmed etiological diagnosis via polymerase chain reaction (PCR) testing of bronchoalveolar lavage fluid (BALF) and/or nasopharyngeal swab samples for respiratory viruses and bacteria. Pediatric septic shock was identified using the international consensus criteria for pediatric sepsis and septic shock (11, 12). The present study was approved by the Medical Ethics Committee of Maternal and Child Health Hospital of Hubei Province (approval no. 2024IEC040). In accordance with the institutional policy that permits the retrospective use of anonymized clinical data for research purposes, the requirement for obtaining patients' informed consent was waived for this study. All patient data were deidentified and handled in strict compliance with the ethical guidelines.

### 2.2 Exclusion criteria

Cases were excluded based on: (1) Neonates and preterm infants; (2) Chronic respiratory or systemic diseases; (3) Congenital/genetic disorders impacting respiratory/systemic functions; (4) Pathogen coinfections (bacterial, fungal, *Mycoplasma spp.*, *Chlamydia spp.*); (5) Extrapulmonary infections; (6) Coinfections with cytomegalovirus, Epstein–Barr virus, or SARS-CoV-2; (7) Cases classified as hospital-acquired infections.

### 2.3 Respiratory virus detection

The nasopharyngeal secretion and BALF samples collected within 24 h of admission were analyzed. Respiratory pathogens, including human rhinovirus, human adenovirus, respiratory syncytial virus (RSV), human parainfluenza virus, human metapneumovirus (hMPV), human bocavirus (HBoV), human coronaviruses (HCoV), influenza A/B, and atypical bacteria (*Mycoplasma spp.*, *Chlamydia spp.*), were detected using multiplex PCR kits. The BALF cultures with microbial identification were carried out according to standard protocols.

### 2.4 Microbial culture and identification

Bacterial identification was performed on the BALF samples collected from patients with suspected pneumonia within 24 h of admission. The samples were inoculated onto blood, chocolate, and MacConkey agar plates. The blood and chocolate agar plates were incubated in a 5%–10% CO_2_ atmosphere for up to 72 h, whereas the MacConkey agar plates were incubated aerobically at 35°C–37°C for 24–48 h. Microbial species in positive cultures were identified by matrix-assisted laser desorption ionization–time of flight mass spectrometry (Bruker Daltonik GmbH, Germany).

### 2.5 Statistical analyses

Statistical analyses were conducted using SPSS version 24.0. Group comparisons of the categorical variables were performed using *χ*^2^ tests with continuity correction for low-frequency cells. For pathogen-specific risk assessment of septic shock, univariate logistic regression models were employed, with each respiratory virus utilized as the sole independent variable and the development of septic shock as the dependent variable. No additional covariates (e.g., age, sex, or comorbidities) were included in the models, as the age distribution varies inherently across the different viral infections, and adjusting for age might obscure the virus-specific pathogenic characteristics in natural infection settings. A *p-value* of <0.05 was considered statistically significant. Given the multiple comparisons across different pathogens, Bonferroni correction was applied to adjust for type I error, with the corrected significance threshold set at *p* < 0.0056 (0.05/9, corresponding to the number of viral pathogens analyzed).

## 3 Results

**Figure 1:**
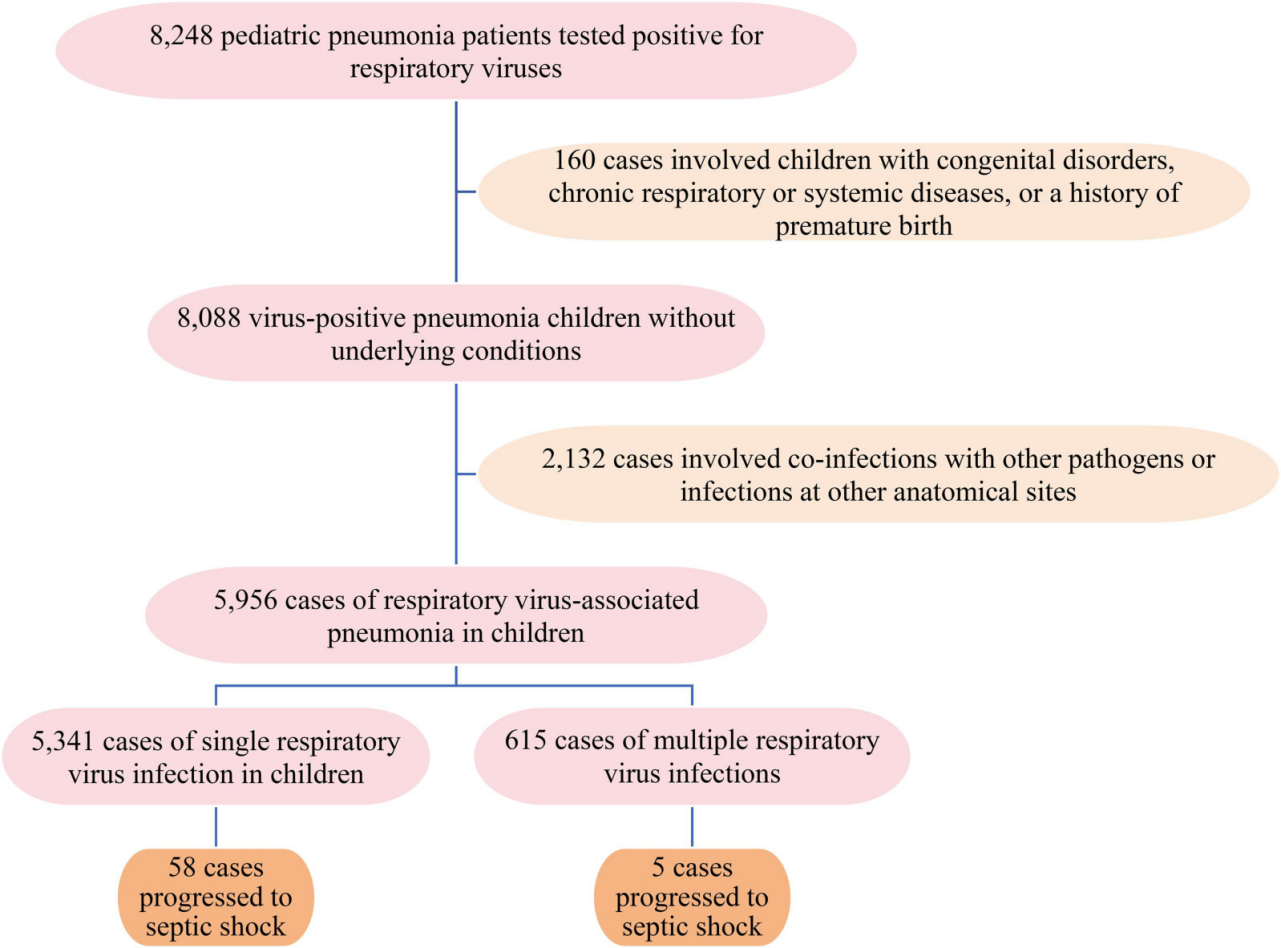
Flowchart of the studied population.

3.1 A total of 5,956 pediatric patients (age range: 1 month to 15 years; median, 3 years; interquartile range, 1–4 years) hospitalized due to viral pneumonia were enrolled in the present study (Figure 1). The cohort comprised 3,479 boys and 2,477 girls, yielding a male-to-female ratio of 1.4:1.
3.2 The incidence of septic shock differs significantly among children with pneumonia, stratified by causative viral respiratory infections (*p* < 0.001). As shown in Table 1, FluA showed the highest incidence rate at 3.70% (14/378), followed by HBoV at 1.57% (2/127) and RSV at 2.24% (29/1,295). The patients with HCoV infections did not develop septic shock. Viral codetection was present in 0.81% of cases (5/615), with an overall incidence of septic shock of 1.06% (63/5,956) across all viral pneumonia cases.
3.3 Logistic regression analysis identified three respiratory viruses associated with the development of septic shock. After Bonferroni correction for multiple comparisons (threshold *p* < 0.0056), only FluA and RSV remained significant independent predictors. FluA showed the strongest association [odds ratio [OR] = 7.506, 95% confidence interval [CI]: 3.872–14.552, *p* < 0.001], followed by RSV (OR = 4.559, 95% CI: 2.584–8.042, *p* < 0.001). HBoV demonstrated a nominal association that was not significant after correction (OR = 3.440, 95% CI: 1.020–11.599, *p* = 0.046). The complete data are presented in Table 2.
3.4 Among the 63 viral infection cases, 55 patients (87.3%) developed shock within 0–3 days of admission, whereas with the remaining eight patients (12.7%) had onset of symptoms within 1 week. In the bacterial group (*n* = 46), 40 patients (87.0%) presented with shock symptoms within 0–3 days of admission, whereas six patients (13.0%) developed shock within 1 week. In the bacterial group, the causes of infections predominantly included *Streptococcus pneumoniae* (*n* = 17), *Pseudomonas aeruginosa* (*n* = 9), *Haemophilus influenzae* (*n* = 7), *Klebsiella pneumoniae* (*n* = 5), *Moraxella catarrhalis* (*n* = 4), and *Staphylococcus aureus* (*n* = 4).
3.5 Table 3 presents a comprehensive comparative analysis of the clinical characteristics and outcomes among pediatric septic shock patients stratified by pathogen type (bacterial, viral, FluA, and RSV). The analysis of clinical characteristics revealed several significant differences between the etiological groups. The age distribution varied significantly, with the FluA group showing a markedly lower proportion of infants aged <1 year than the bacterial group (7.1% vs. 47.8%, *p* = 0.023). Conversely, the RSV group had a significantly higher proportion of infants aged <1 year than the FluA group (69.0% vs. 7.1%, *p* < 0.001).

The respiratory manifestations also differed. The incidence of respiratory failure/acute respiratory distress syndrome (ARDS) was significantly higher in the overall viral group than in the bacterial group (88.9% vs. 52.2%, *p* < 0.001). This difference was also significant specifically between the RSV and bacterial groups (75.9% vs. 52.2%, *p* = 0.040).

**Table 1:** Incidence of septic shock in hospitalized children with viral pneumonia.

Detected respiratory viruses	Viral pneumonia	Septic shock	Incidence (%)
HRV	1,361	4	0.29
HAdV	436	2	0.46
RSV	1,295	29	2.24
HPIV	641	4	0.62
hMPV	907	2	0.22
HBoV	127	2	1.57
HCoV	49	0	0.00
Flu A	378	14	3.70
Flu B	147	1	0.68
Viral codetection	615	5	0.81
Total	5,956	63	1.06

HRV, human rhinovirus; HAdV, human adenovirus; RSV, respiratory syncytial virus; HPIV, human parainfluenza viruses; hMPV, human metapneumovirus; HBoV, human bocavirus; HCoV, human coronaviruses; FluA/B, influenza viruses A/B.

**Table 2:** Association between respiratory viruses and septic shock: multivariable logistic regression results.

Pathogen	Coefficient (B)	*p-value* (Original)	*p-value* (Bonferroni corrected)	OR (95%CI)
Flu A	2.016	<0.001	<0.0056	7.506 (3.872–14.552)
RSV	1.517	<0.001	<0.0056	4.559 (2.584–8.042)
HBoV	1.235	0.046	0.414	3.440 (1.020–11.599)

The Bonferroni correction was applied with a significance threshold of 0.0056 (0.05/9 pathogens). Wide confidence intervals for HBoV indicate potential uncertainty due to limited sample size.

CI, confidence interval; OR, odds ratio.

**Table 3:** Comparative analysis of clinical characteristics and outcomes in pediatric septic shock.

Characteristics	Bacterial infection (*n* = 46)	Viral infection (*n* = 63)	*p-value* (Bact vs. Viral)	Flu A infection (*n* = 14)	*p-value* (Bact vs. FluA)	RSV infection (*n* = 29)	*p-value* (Bact vs. RSV)	*p-value* (FluA vs. RSV)
1. Demographics								
Male gender, *n* (%)	30 (65.2)	33 (52.4)	0.180	7 (50.0)	0.305	18 (62.1)	0.782	0.452
Age								
<1, *n* (%)	22 (47.8)	31 (49.2)	0.708	1 (7.1)	0.023	20 (69.0)	0.098	<0.001
1–5, *n* (%)	20 (43.5)	29 (46.0)		11 (78.6)		9 (31.0)		
≥6, *n* (%)	4 (8.7)	3 (4.8)		2 (14.3)		0 (0)		
2. Respiratory								
PaO_2_:FIO_2_ ≤ 200 and IMV, *n* (%)	36 (78.3)	49 (77.8)	0.952	10 (71.4)	0.866	21 (72.4)	0.564	1.000
Respiratory Failure/ARDS	24 (52.2)	56 (88.9)	<0.001	11 (78.6)	0.149	22 (75.9)	0.040	1.000
Support Measures								
HFNC, *n* (%)	5 (10.9)	7 (11.1)	0.998	1 (7.1)	1.000	5 (17.2)	0.727	0.852
CPAP/NIV, *n* (%)	5 (10.9)	7 (11.1)		1 (7.1)		3 (10.3)		
IMV, *n* (%)	36 (78.3)	49 (77.8)		12 (85.7)		21 (72.4)		
3. Cardiovascular								
Hyperlactatemia, *n* (%)	13 (28.3)	9 (14.3)	0.073	5 (35.7)	0.594	2 (6.9)	0.05	0.050
Age-adjusted hypotension, *n* (%)	1 (2.2)	5 (7.9)	0.380	0 (0)	1.000	1 (3.4)	1.000	1.000
≥2 Vasoactive medications, *n* (%)	27 (58.7)	37 (58.7)	0.997	10 (71.4)	0.586	16 (55.2)	0.764	0.491
Inotropic agents used, *n* (%)	34 (60.9)	28 (54.0)	0.472	3 (21.4)	0.014	18 (62.1)	0,917	0,022
Refractory shock, *n* (%)	4 (8.7)	11 (17.5)	0.263	6 (42.9)	0.007	0 (0)	0.154	<0.001
4. Coagulation								
Platelets <100 × 10^3^/µL, *n* (%)	6 (13.0)	6 (9.5)	0.562	3 (21.4)	0.732	1 (3.4)	0.325	0.180
INR >1.3/D-dimer >2 mg/L/Fibrinogen100< mg/dL	28 (60.9)	28 (44.4)	0.090	12 (85.7)	0.161	10 (34.5)	0.026	0.005
5. Adjunct Therapies								
IVIG administration, *n* (%)	27 (58.7)	39 (61.9)	0.735	8 (57.1)	0.918	22 (75.9)	0.128	0.210
Systemic steroids, *n* (%)	18 (39.1)	11 (17.5)	0.011	1 (7.1)	0.026	8 (27.6)	0.306	0.231
6. Neurological								
Glasgow Coma Scale score ≤10	4 (8.7)	8 (12.7)	0.727	5 (35.7)	0.040	3 (10.3)	1.000	0.113
7. Outcomes								
28-day mortality-Survived	39 (84.8)	47 (74.6)	0.198	9 (64.3)	0.093	23 (79.3)	0.542	0.290
28-day mortality-Died/Unknown	7 (15.2)	16 (25.4)		5 (35.7)		6 (20.7)		
Length of stay in patients with routine discharge (days), mean ± SD	24.21 ± 18.289	17.32 ± 9.229	0.026	18.67 ± 8.930	0.384	16.52 ± 9.229	0.066	0.556

Unknown, Withdrawal of treatment or interfacility transfer.

Bact, bacterial; IMV, invasive mechanical ventilation; ARDS, acute respiratory distress syndrome; HFNC, high-flow nasal cannula; CPAP, continuous positive airway pressure; NIV, non-invasive ventilation; INR, international normalized ratio of prothrombin time; IVIG, intravenous immunoglobulin.

Notable differences in the cardiovascular and metabolic parameters were also observed. A trend toward a higher rate of hyperlactatemia was observed in the RSV group than in the bacterial group (6.9% vs. 28.3%, *p* = 0.050). Regarding the management and outcomes, the use of inotropic agents (including digoxin, dopamine hydrochloride, dobutamine hydrochloride, and milrinone) was significantly less frequent in the FluA group than in the bacterial (21.4% vs. 60.9%, *p* = 0.014) and RSV (21.4% vs. 62.1%, *p* = 0.022) groups. Furthermore, the incidence of refractory shock was significantly higher in the FluA group than in the bacterial (42.9% vs. 8.7%, *p* = 0.007) and RSV (42.9% vs. 0.0%, *p* < 0.001) groups. The coagulation profiles also showed significant variations. The prevalence of coagulopathy (defined as INR > 1.3, D-dimer > 2 mg/L, or fibrinogen < 100 mg/dL) was significantly lower in the RSV group than in the bacterial (34.5% vs. 60.9%, *p* = 0.026) and FluA (34.5% vs. 85.7%, *p* = 0.005) groups.

The type of adjunct therapy used differed significantly, with systemic steroids being administered less frequently in the overall viral group compared to the bacterial group (17.5% vs. 39.1%, *p* = 0.011). This difference was also significant between the FluA and bacterial groups (7.1% vs. 39.1%, *p* = 0.026).

Significant neurological compromise (Glasgow Coma Scale score of ≤10) was more frequently observed in the FluA group compared to the bacterial group (35.7% vs. 8.7%, *p* = 0.040).

Finally, the outcomes analysis showed a significantly shorter mean hospital stay in the viral group than in the bacterial group (17.32 ± 9.229 vs. 24.21 ± 18.289 days, *p* = 0.026).

## 4 Discussion

To the best of our knowledge, no prior studies have compared the rates of septic shock among patients with common viral respiratory infections. In our study, we found that 1.06% (63 out of 5,956) of patients with viral pneumonia experienced septic shock, a rate consistent with the current epidemiological data (13, 14). The occurrence of septic shock varied considerably depending on the specific pathogen responsible for the infections. Among the different pathogens studied, patients with FluA infections had the highest rate of septic shock, with 3.70% of cases developing severe complications. Contrarily, the infections caused by hMPV exhibited a lower septic shock rate, with only 0.22% of cases progressing to septic shock. Interestingly, no case of septic shock was observed in the 49 children infected with HCoV, suggesting that this particular pathogen was not associated with this severe outcome in the studied cohort. These results emphasize the importance of developing clinical surveillance systems tailored to individual pathogens. We recommend the following three primary research priorities: (1) multicenter validation of pathogen-specific incidence thresholds, (2) developing models of viral load kinetics to pinpoint the critical risk periods, and (3) conducting mechanistic comparisons of systemic inflammation between different viruses.

The results of the present study suggest that children with viral pneumonia, particularly that caused by FluA or RSV, may be at an increased risk of developing septic shock. FluA infection triggers an excessive release of proinflammatory cytokines (e.g., IL-6, TNF-α, and IFN-γ), leading to a “cytokine storm,” a hallmark of systemic inflammation (15, 16). In coculture models, FluA-infected lung epithelial cells release inflammatory mediators that enhance proinflammatory cytokine secretion by endothelial cells, exacerbating systemic inflammation (16). Unlike FluA (Th1-dominant), RSV often skews toward Th2 polarization, which may exacerbate mucus production, airway obstruction, and prolonged inflammation, indirectly promoting systemic immune activation (17). HBoV immunology remains understudied, although emerging evidence highlights CD4^+^ T cells as key mediators of the antiviral response (18, 19). Our analysis found an association between HBoV infection and septic shock (OR = 3.44, 95% CI: 1.02–11.59), but this finding should be interpreted cautiously due to the wide CI observed—likely a result of the small sample size–and loss of statistical significance after Bonferroni correction. These findings suggest a potential link that requires validation in larger cohorts to clarify how HBoV modulates CD4^+^ T cell-mediated immunity to drive disease severity. Future studies should focus on delineating the specific mechanisms by which these viruses contribute to the development of septic shock. This includes investigating the temporal dynamics of cytokine release, role of immune cell subsets, and potential for therapeutic interventions that could modulate the inflammatory response. Understanding these pathways could ultimately lead to improved management strategies for pediatric pneumonia and its complications.

A critical finding of our study is that children ultimately diagnosed with bacterial or viral septic shock presented with equally severe and overlapping early admission symptoms, including high fever, cough, and respiratory distress that rapidly progressed to respiratory failure. This clinical similarity underscores the impossibility of reliably differentiating etiology based solely on initial symptomatology. It also explains why both groups required comparable urgency for intensive care admission and exhibited no significant difference in 28-day mortality.

The significantly higher incidence of respiratory failure/ARDS in viral infections than in bacterial infections may reflect the distinct pathophysiological mechanisms inherent to viral pathogens. Although the cytokine profiles are specific to each virus, a shared characteristic of all recent pandemic viruses is their ability to provoke an excessive early cytokine response (17–20). Pulmonary endothelial cells play a vital role in managing the recruitment of innate immune cells and production of cytokines and chemokines during H1N1 infections (21). This leads to speculation about whether a similar process occurs in ARDS triggered by other respiratory viruses. Unlike this viral-driven mechanism, in bacterial pneumonia, respiratory failure occurs through the combined effects of surfactant disruption, inflammation-related barrier damage, airway obstruction, and thromboxane A2-induced narrowing of the pulmonary blood vessels (22–24). In this study, we observed that children with viral-associated septic shock had a significantly shorter length of hospital stay compared to the bacterial group (17.3 vs. 24.2 days, *p* = 0.026). This disparity must be interpreted cautiously, as it is likely confounded by differing treatment requirements. Bacterial infections typically necessitate prolonged, completed courses of intravenous antibiotics, often mandating continued hospitalization. In contrast, care for viral infections is primarily supportive, and discharge can be facilitated once the need for respiratory support resolves (25). Thus, the observed difference may reflect these contrasting treatment paradigms rather than a direct measure of intrinsic disease severity.

The contrast in age distribution between the bacterial and FluA groups, particularly the smaller proportion of infants aged <1 year in the FluA cases, emphasizes the unique age-related susceptibility patterns of viral and bacterial infections. Bacterial pathogens, such as *S. pneumoniae*, show predilection for infants, consistent with the peaks in pneumococcal nasopharyngeal colonization and anatomical vulnerabilities during early childhood (26). Conversely, FluA's epidemiological pattern favors transmission among older children, where heightened group exposure facilitates viral spread (27).

The present study also revealed a significantly higher incidence of severe neurological impairment in pediatric patients with septic shock associated with FluA infections than in the bacterial cades, suggesting distinct central nervous system-specific pathophysiological mechanisms. FluA infections in children, particularly those aged <5 years who are unvaccinated, induce neurological damage such as encephalitis with seizures and altered consciousness, highlighting its neuroinvasive potential even in previously healthy individuals. Neuroimaging reveals diverse brain injury patterns, implicating viral or immune-mediated mechanisms, alongside elevated AST/LDH indicating a multisystem involvement (28, 29). Contrarily, neurological compromise in patients with bacterial pneumonia arises via pneumolysin (PLY)-mediated nasal epithelial barrier disruption, enabling direct brain invasion; PLY-driven neuroinflammation; and hyperglycemia-induced blood–brain barrier dysfunction with oxidative stress (30, 31).

Pediatric patients with FluA infections exhibit a significantly higher incidence of refractory shock (42.9%) than those with bacterial infections (8.7%, *p* = 0.007), a discrepancy rooted in the distinct immunopathological mechanisms of FluA relative to bacterial sepsis. FluA triggers a rapid activation of the innate immune pathways, such as NF-κB and STAT1/3, leading to an early and intense surge in proinflammatory cytokines, including TNF-α, IL-6, and IL-1β, that surpasses both the speed and magnitude of responses observed in bacterial sepsis (32). Concurrently, the virus activates multiple cell death pathways—pyroptosis, apoptosis, and necroptosis—leading to massive alveolar damage and further cytokine release; this pattern is absent in bacterial infections that typically induce slower inflammation via TLR signaling without synchronous multipathway cell death (33, 34). Although neuraminidase inhibitors reduce viral replication, they have limited efficacy against established cytokine storms or tissue damage, unlike bacterial sepsis, which more often responds to antibiotic treatment and supportive care (35). This rapid, intense, and multifaceted activation of immune and cell death pathways in FluA infections underlies its more severe, treatment-refractory shock phenotype compared to bacterial-associated septic shock. Septic shock in children with RSV infections, conversely, is believed to result from virus-induced hyperinflammatory responses, immunosuppression, and oxidative stress (36, 37). These pathophysiological features may explain the absence of refractory shock in the RSV group (0%, *p* < 0.001 vs. FluA group), highlighting a key mechanistic distinction among viral etiologies.

The significantly higher prevalence of coagulation abnormalities in FluA patients (85.7% vs. 34.5% in RSV patients) underscores a potential association between FluA infection and hematologic dysregulation. A previous multicenter cohort study involving 528 outpatients and 209 hospitalized patients demonstrated that the third tertile of D-dimer (>0.5 mg/L) was significantly associated with clinical deterioration or death within 14 days in hospitalized patients (OR = 3.2). This association was more pronounced in severe FluA cases, with 53.8% of the ICU patients exhibiting D-dimer levels of >2 mg/L, positively correlating with a multiorgan failure risk (36). The underlying mechanisms likely involve virus-induced endothelial damage and coagulation-fibrinolysis imbalance. FluA infection triggers cytokine storms, promoting tissue factor expression and activating the extrinsic coagulation pathway (37). Viral hemagglutinin may directly activate platelets, leading to microvascular thrombosis and subsequent compensatory hyperfibrinolysis, which manifests as markedly elevated D-dimer levels (38, 39).

In a recent multicenter retrospective cohort study on viral pathogen-induced septic shock involving 1,247 children with septic shock, 305 viral infections (149 viral respiratory infections) were identified (7). Univariate logistic regression showed that the viral respiratory infections were associated with higher incidences of respiratory failure and disseminated intravascular coagulation (DIC), prolonged invasive mechanical ventilation, and increased use of immunoglobulins and antiviral agents. Compared to the viral nonrespiratory groups, viral respiratory infections had significantly higher rates of respiratory failure/ARDS, DIC, and immunoglobulin administration (40). For bacterial pathogen-related septic shock, previous studies have indicated temporal and regional variations in the dominant pathogens. A large-scale analysis using the Kids' Inpatient Database revealed methicillin-resistant *S. aureus* had the highest mortality (14.42%) among the gram-positive bacteria, whereas *P. aeruginosa* showed a striking 21.49% mortality rate among the gram-negative bacteria—a 2.58-fold higher than the other pathogens (41). Contrarily, the present study uniquely identifies FluA and RSV as key viral pathogens in pediatric pneumonia-associated septic shock. We systematically compared their clinical profiles with those of bacterial-induced shock, highlighting distinct differences in hospitalization durations, complication rates, and pathogen-specific outcomes, which underscores the unique pathophysiology of viral-associated septic shock in childhood pneumonia.

Based on these insights, our study findings argue for integrating rapid multiplex respiratory viral PCR testing into the initial workup for pediatric cases of sepsis. This would allow clinicians to confidently initiate antibiotic de-escalation when a viral pathogen is identified, or even consider discontinuation entirely when bacterial coinfection is deemed unlikely and clinical stability is achieved. This approach, supported by evidence on biomarker-guided stewardship, promotes a more precise management strategy that reduces unnecessary antibiotic exposure while maintaining patient safety (25).

Our study has several limitations that warrant consideration. First, its retrospective design may have introduced epidemiological bias, as empirical antimicrobial treatment in blood culture-negative cases could have hindered comprehensive pathogen identification. Second, although methodologically necessary, the exclusion of mixed viral–bacterial or viral–*Mycoplasma* coinfections introduces selection bias and limits the applicability of our findings to real-world clinical scenarios, where such coinfections are frequently encountered. This design also prevents the evaluation of potential synergistic effects on the progression of septic shock. Third, despite spanning a three-year period, the number of viral-associated septic shock cases remained limited, resulting in small sample sizes for individual viral subgroups and reduced statistical power. For instance, subgroup analyses for Influenza A (*n* = 14) and RSV (*n* = 29) are underpowered and should be interpreted as descriptive and hypothesis-generating rather than definitive. Fourth, some cases of septic shock may have been caused by undetected or unidentified pathogens, reflecting current limitations in diagnostic capabilities. Lastly, our data were derived from two hospitals within a single metropolitan area. While this ensures consistency in clinical management, it may restrict the external validity and generalizability of our findings to other geographic regions or healthcare systems with differing patient demographics and resource availability.

Nevertheless, our study findings retain clinical relevance, as they establish the baseline epidemiological patterns for pediatric viral pneumonia complications in our region. However, future multicenter collaborations with prospective designs and larger sample sizes are still warranted to validate these preliminary associations and refine the risk stratification models.

## 5 Conclusion

In the present research, FluA and RSV infections were identified as the primary risk factors for septic shock in children with viral pneumonia, which presents distinctly from bacterial-associated septic shock. Integrating rapid multiplex viral PCR testing into the initial sepsis workup may facilitate early antibiotic de-escalation upon confirmation of a viral cause, promoting antimicrobial stewardship and reducing unnecessary exposure among patients.

## Data Availability

The original contributions presented in the study are included in the article/Supplementary Material, further inquiries can be directed to the corresponding author.
